# Enhanced Expression of Markers for Astrocytes in the Brain of a Line of GFAP-TK Transgenic Mice

**DOI:** 10.3389/fnins.2017.00212

**Published:** 2017-04-12

**Authors:** Xiaoqin Zhang, Dongpi Wang, Hongyu Pan, Binggui Sun

**Affiliations:** ^1^Department of Neurobiology, Key Laboratory of Medical Neurobiology (Ministry of Health of China), Key Laboratory of Neurobiology of Zhejiang ProvinceHangzhou, China; ^2^Children's Hospital, Zhejiang University School of MedicineHangzhou, China

**Keywords:** astrocytes, GFAP-TK mice, adult neurogenesis, GFAP, vimentin

## Abstract

GFAP-TK mice are widely used in studies on neurogenesis and reactive astrocytes. Previous studies reported that GCV treatment in GFAP-TK mice resulted in reduced neurogenesis and deletion of proliferating GFAP-expressing astrocytes without affecting mature GFAP-expressing astrocytes. In the present study, we found that GFAP- and vimentin-expressing astrocytes were dramatically increased in the cortex and hippocampus with or without GCV treatment in a line of GFAP-TK mice (Jackson Laboratory, Stock No. 005698), while the neurons and microglia were not affected. In a second line of GFAP-TK mice (MMRRC, Stock No. 037351-UNC) generated in Dr. Heather Cameron's laboratory in NIH, however, no difference in GFAP and vimentin expression was found in both hippocampus and cortex, regardless of GCV treatment or not. Furthermore, enhanced expression of aquaporin 4 (AQP4) was found in the cortex and hippocampus of the GFAP-TK mice from Jackson lab but not in the brain of GFAP-TK mice from NIH. Our data suggested that we should be careful to select different lines of GFAP-TK mice to study adult neurogenesis or reactive astrocytes.

## Introduction

New neurons could be continuously generated in the hippocampus of adult mammals (Spalding et al., 2013; Bond et al., 2015; Kempermann et al., 2015; Bonfanti, 2016). The newborn neurons integrate into the pre-existing neural circuits (Van Praag et al., 2002; Toni et al., 2008; Vivar et al., 2012; Restivo et al., 2015; Sultan et al., 2015; Toni and Schinder, 2016) and are associated with several cognitive functions such as learning and memory, pattern separation, and mood regulation (Clelland et al., 2009; Deng et al., 2010; Ming and Song, 2011; Sahay et al., 2011; Gu et al., 2012; Christian et al., 2014). Abnormal generation of new neurons in the adult hippocampus was also implicated in neurodegenerative disorders, epilepsy, and depression (Scharfman and Hen, 2007; Chen et al., 2008; Demars et al., 2010; Snyder et al., 2011). Therefore, it is of specially interesting to investigate whether manipulating the generation of new born neurons in the adult brain could affect the pathogenesis of different neurological disorders.

A variety of methods have been developed to regulate the neurogenesis in adult brain. Enriched environment and physical exercise, for example, could significantly increase the number of newborn neurons in the hippocampus of adult mice or rats (Wolf et al., 2006; Mirochnic et al., 2009; Valero et al., 2011). Intraperitoneal injection of MAM (Methylazoxymethanol acetate), a mitotic inhibitor, however, greatly reduced the number of newborn neurons in the adult hippocampus (Hsiao et al., 2014; Liu et al., 2016). Recently, optogenetics and chemogenetics were also used to regulate the activity of newborn neurons (Temprana et al., 2015). On the other hand, several lines of transgenic mice such as GFAP-TK and nestin-TK mice were generated and they have been used together with ganciclovir (GCV) treatment to inhibit adult neurogenesis (Singer et al., 2009; Snyder et al., 2011; Cho et al., 2015). GFAP is a marker of adult neural stem cells and astrocytes. Therefore, both astrocytes and neural stem cells could potentially be affected when GFAP-TK mice were used in different studies.

In the present study, we compared the expression of several glial and neuronal markers between two lines of GFAP-TK mice with or without GCV treatment. We found that adult neurogenesis was effectively inhibited in both lines of GFAP-TK mice after GCV treatment. However, the expression of GFAP and vimentin was dramatically increased in the cortex and hippocampus of one GFAP-TK line but not in the other. Our results suggested that we should be careful to select different lines of GFAP-TK mice to study adult neurogenesis or reactive astrocytes.

## Materials and methods

### Animals

The first line of GFAP-TK mice (TK-1) were purchased from the Jackson Laboratory (Stock No. 005698). These mice were on a mixed C57BL/6J; C57BL/6N genetic background, and transgene-derived HSV-TK in these mice was present exclusively in cells expressing endogenous *Gfap* (Bush et al., 1998; Garcia et al., 2004). The second line of GFAP-TK mice (Snyder et al., 2011) (TK-2) (MMRRC, Stock No. 037351-UNC) were provided by Dr. Tianming Gao (Southern Medical University, Guangzhou, China) with permission from Dr. Heather Cameron (Section of Neural Plasticity, NIMH/NIH). These mice were bred on a mixed C57Bl/6:CD-1 background. All mice were housed under standard conditions at 22°C and a 12 h light: dark cycle with free access to food and water. The study as well as all experimental protocols were reviewed and approved by the Institutional Animal Care and Use Committee of the Zhejiang University.

### Drug treatment

For ganciclovir (GCV, Roche; in 0.9% sterile saline) treatment, osmotic mini-pumps (Model 2004; Alzet; 0.25 μl/h release rate) containing different dosage of GCV (0, 10, 20, 40 mg kg^−1^ per day) were implanted subcutaneously in the back of mice (2.5 months old) after anesthetization with 1% pentobarbital sodium. Mice were treated with GCV for 4 weeks.

### Immunostaining

Animals were perfused transcardially with ice-cold saline and brains were removed and immersed into 4% PFA in PBS. After dehydration in 30% sucrose in PBS, coronal sections (30 μm in thickness, one in tenth series) were prepared with a sliding microtome (Leica). For immunofluorescence staining, free floating sections were incubated with the following primary antibodies: goat anti-DCX (sc-8066, Santa Cruz; 1:100), rabbit anti-Ki67 (s2532, Sigma; 1:500), rabbit anti-Iba1 (019-19741, Wako; 1:1,000), rabbit anti-NeuN (MABN140, Millipore; 1:1,000), rabbit anti-MAP2 (4542, Cell Signaling; 1:500), rabbit anti-AQP4 (AQP-004, Alomone labs; 1:1,000) followed by incubation with appropriate secondary antibodies conjugated with 488 (Vector Laboratories, 1:500) or Cy3 or 594 (Jackson, 1:500). For immunohistochemical staining, brain sections were incubated with 3% H_2_O_2_ in methanol, to quench endogenous peroxidase activity followed by incubation with primary antibodies: mouse anti-GFAP (G3893, Sigma; 1:2,000) and rabbit anti-vimentin (ab92547, abcam; 1:1,000). After washing, sections were incubated with biotinylated goat anti-mouse or rabbit (1:200, Vector Laboratories). Binding of the antibodies was detected with the Elite kit (Vector Laboratories) with diaminobenzidine (Sigma) and H_2_O_2_ for development. All immunostaining analyses were done blindly.

### Quantification

For cell counting, images were obtained with digital camera (Olympus BX53, Japan). The numbers of GFAP-positive and vimentin-positive cells in the cortex were determined by counting positive cells in two areas (400 × 400 μm) of each section in every 10th serial coronal section throughout the rostrocaudal extent of the cortex. The numbers of GFAP-positive cells were determined by counting positive cells in the DG, hilus, CA3, and CA1–2 of the hippocampus. The numbers of GFAP-positive and vimentin-positive cells were normalized to the cross-sectional area of the region involved and expressed per mm^2^. At least three coronal sections were analyzed per mouse, and the average of the individual measurements was used to calculate group means. The DCX-positive cells and Ki67-positive cells from every 10th section covering the entire area of the DG were counted with a fluorescence microscope (Olympus BX53, Japan) with a × 40 objective. At least five sections from each side of the DG were counted per animal. The number of animals in each group was indicated in the figure legends.

For quantification of Iba1, NeuN, MAP2, and AQP4, three coronal sections (300 μm apart) per mouse were selected. Optical density was determined with image analysis software and averaged in two areas (0.16 mm^2^ each) of the cortex or of the hippocampus. The fluorescence intensity was quantified as the mean gray value of the same section. The number of animals in each group was indicated in the figure legends.

### Western blotting analysis

Mouse cortex or hippocampal samples were homogenized in RIPA buffer containing 10 mM HEPES (pH 7.4), 150 mM NaCl, 50 mM NaF, 1 mM EDTA, 1 mM dithiothreitol, 1 mM phenylmethylsulfonyl fluoride, 1 mM Na_3_VO_4_, 10 μg/ml leupeptin, 10 μg/ml aprotinin, and 1% SDS. Equal amounts of protein (by BCA assay) were resolved by SDS-PAGE and transferred to nitrocellulose membranes. After blocking, membranes were labeled with rabbit anti-AQP4 (AQP-004, Alomone labs; 1:1,000), mouse anti-GFAP (G3893, Sigma; 1:5,000), rabbit anti-vimentin (ab92547, abcam; 1:1,000), goat anti-Iba1 (016-20001, Wako; 1:200), or mouse anti-GAPDH antibody (sc-137179, Santa Cruz; 1:10,000) and incubated with HRP-goat anti-rabbit antibody (GAR007, LiankeBio; 1:5,000) or goat anti-mouse antibody (GAM007, LiankeBio; 1:5,000). Bands were visualized by enhanced chemiluminescence, and the densitometry measurements of the bands were acquired from scanned images with Quantity One software (Bio-Rad).

### Statistical analyses

All data are presented as mean ± SEM. Differences among multiple means with one variable were evaluated by one-way ANOVA and the Tukey-Kramer *post-hoc* test. Differences between two means were assessed with unpaired, two-tailed *t*-test. Only values with *p* < 0.05 were accepted as significant. Statistical analyses were performed with Graphpad Prism 5 (San Diego, CA).

## Results

### Effects of GCV treatment on ablation of adult-born neurons between TK-1 and TK-2 mice

**Figure 1:**
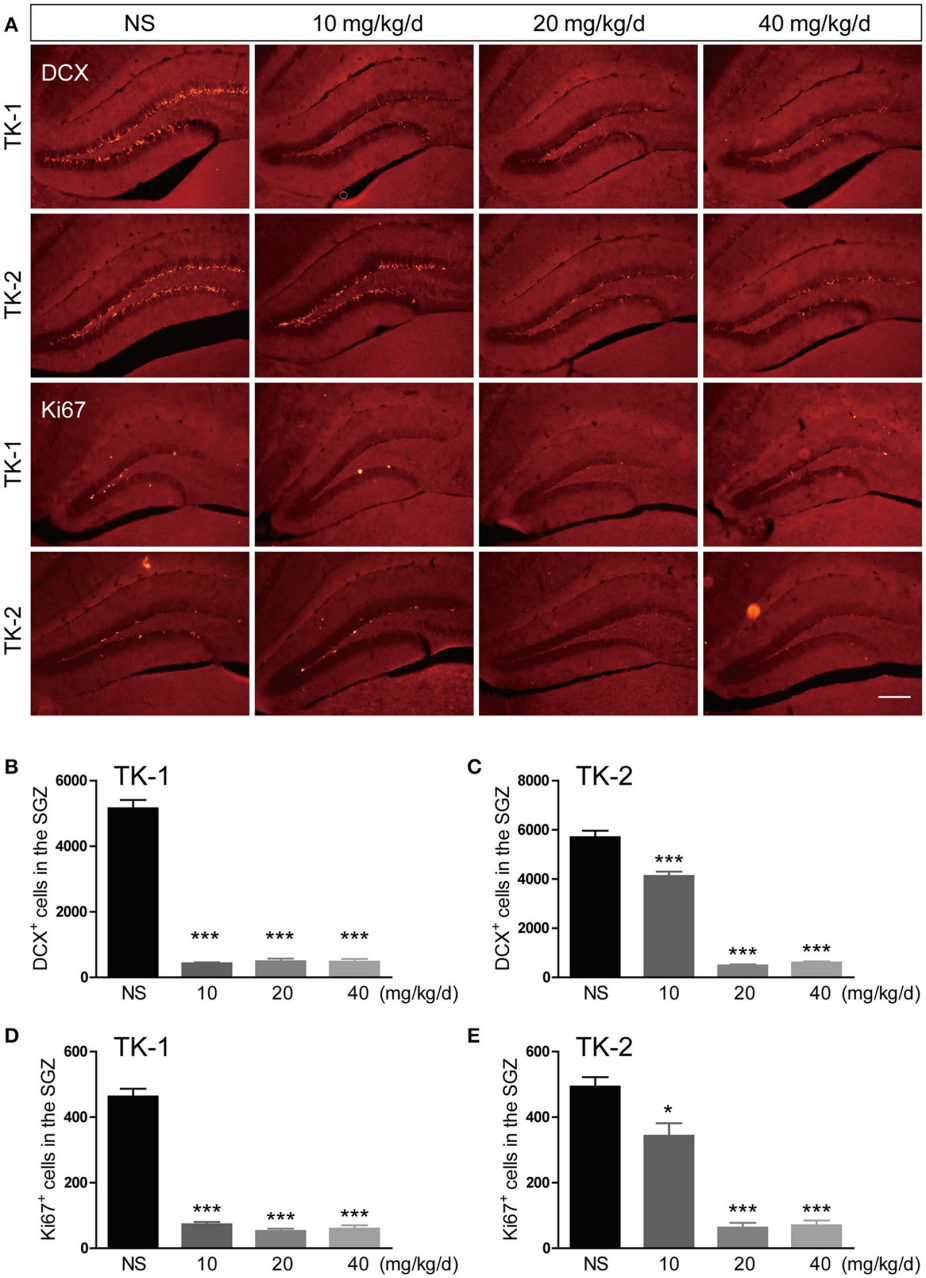
**Effects of GCV treatment on ablation of adult-born neurons in the dentate gyrus of TK-1 and TK-2 mice. (A)** Representative photomicrographs of DCX^+^ cells and Ki67^+^ cells in the dentate gyrus of TK-1 and TK-2 mice with GCV treatment at 0, 10, 20, 40 mg kg^−1^ per day. Scale bar, 200 μm. **(B,C)** Quantification of the number of DCX positive cells in the dentate gyrus of TK-1 mice (*n* = 3 mice, 5 brain slices for each mouse) and TK-2 mice (*n* = 3 mice, 5 brain slices for each mouse) with GCV treatment at 0, 10, 20, 40 mg kg^−1^ per day. **(D,E)** Quantification of the number of Ki67 positive cells in the dentate gyrus of TK-1 mice (*n* = 3 mice, 5 brain slices for each mouse) and TK-2 mice (*n* = 3 mice, 5 brain slices for each mouse) with GCV treatment at 0, 10, 20, 40 mg kg^−1^ per day. Data represent mean ± SEM,^*^P < 0.05, ^***^P < 0.001 (one-way ANOVA with post hoc Turkey's multiple comparison test).

Both TK-1 and TK-2 mice (2.5-month-old) were treated with different dosages of GCV or vehicle for 4 weeks. One week after the completion of GCV treatment, mice were perfused and processed for immunostaining. Results of doublecortin (DCX) staining revealed that the number of adult-born immature neurons in the dentate gyrus was significantly reduced at 10, 20, 40 mg kg-1 per day in TK-1 mice (Figures 1A,B). However, the number of adult-born immature neurons in the dentate gyrus of TK-2 mice was only reduced with GCV treatment at 20, 40 mg kg-1 per day (Figures 1A,C). Similarly, Ki67 staining showed that the number of proliferating neural progenitors was also dramatically reduced in the DG of TK-1 mice with GCV treatment at 10, 20, 40 mg kg-1 per day and of TK-2 mice with GCV treatment at 20, 40 mg kg-1 per day (Figures 1D,E). These data suggested that the sensitivity to GCV treatment on adult-born neurons was different between TK-1 and TK-2 mice.

### The expression of GFAP and vimentin was increased in the cortex and hippocampus of TK-1 mice

**Figure 2:**
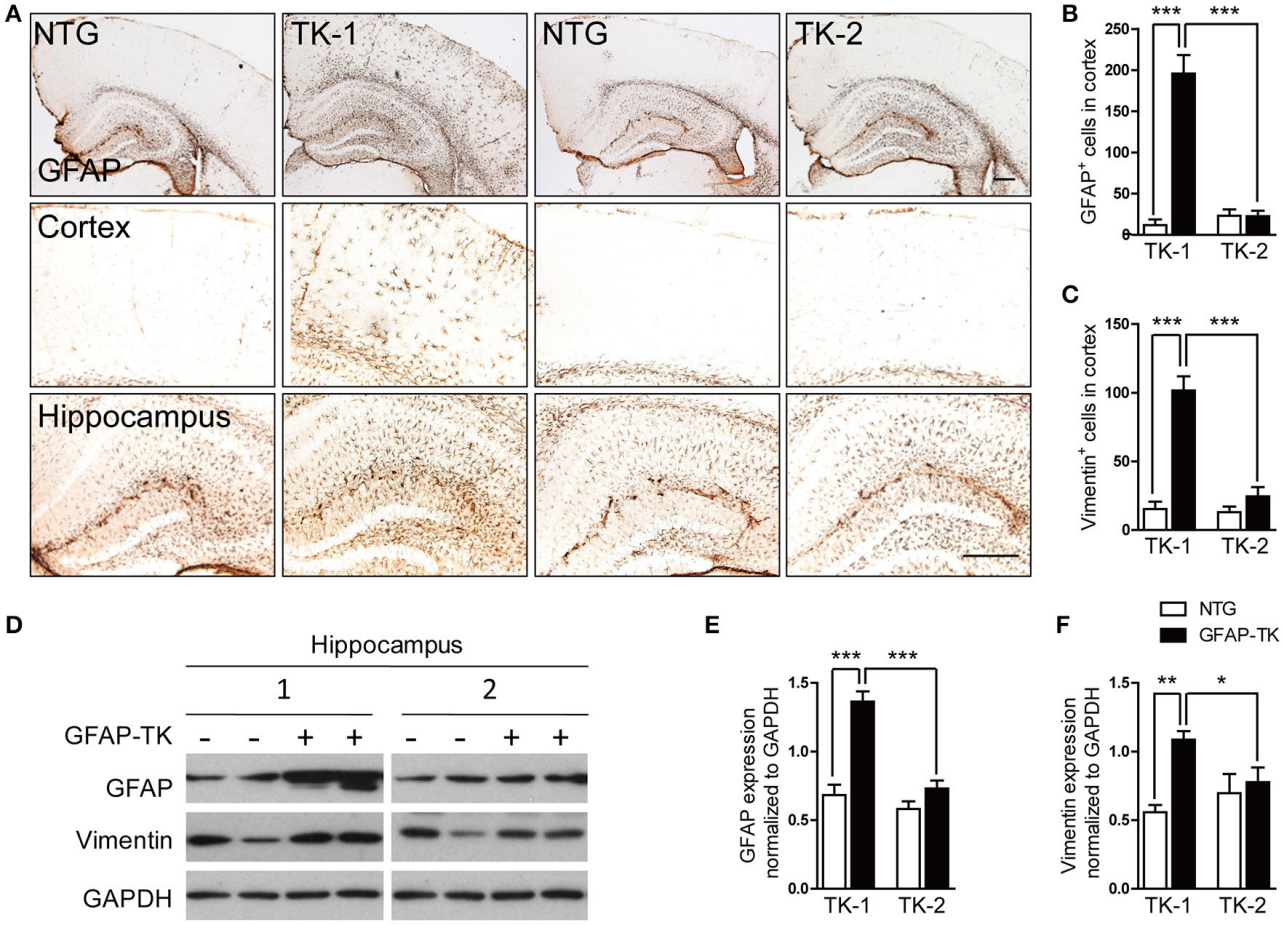
**The expression of GFAP and vimentin was dramatically increased in the hippocampus and cortex of 3-month-old TK-1 mice. (A)** Representative photomicrographs of GFAP^+^ cells in the cortex and hippocampus of 3-month-old TK-1 and TK-2 mice and their age-matched controls. Scale bar, 300 μm. **(B)** Quantification of the number of GFAP^+^ cells in the cortex of 3-month-old TK-1 (*n* = 11 mice, 3 brain slices per mouse) and TK-2 mice (*n* = 8 mice, 3 brain slices per mouse) and their age-matched controls (*n* = 4 mice per group, 3 brain slices per mouse). **(C)** Quantification of the number of vimentin^+^ cells in the cortex of 3-month-old TK-1 (*n* = 11 mice, 3 brain slices per mouse) and TK-2 mice (*n* = 9 mice, 3 brain slices per mouse) and their age-matched controls (*n* = 4 mice per group, 3 brain slices per mouse). **(D)** Protein bands of GFAP and vimentin, GAPDH severed as the loading control. **(E)** Quantification of the levels of GFAP in the hippocampus of 3-month-old TK-1 (*n* = 6 mice) and TK-2 mice (*n* = 5 mice) and their age-matched controls (*n* = 4 mice per group). **(F)** Quantification of the levels of vimentin in the hippocampus of 3-month-old TK-1 (*n* = 7 mice) and TK-2 mice (*n* = 8 mice) and their age-matched controls (*n* = 3 mice per group). Data represent mean ± SEM, ^*^P < 0.05, ^**^P < 0.01, ^***^P < 0.001 (unpaired *t*-test).

**Figure 3:**
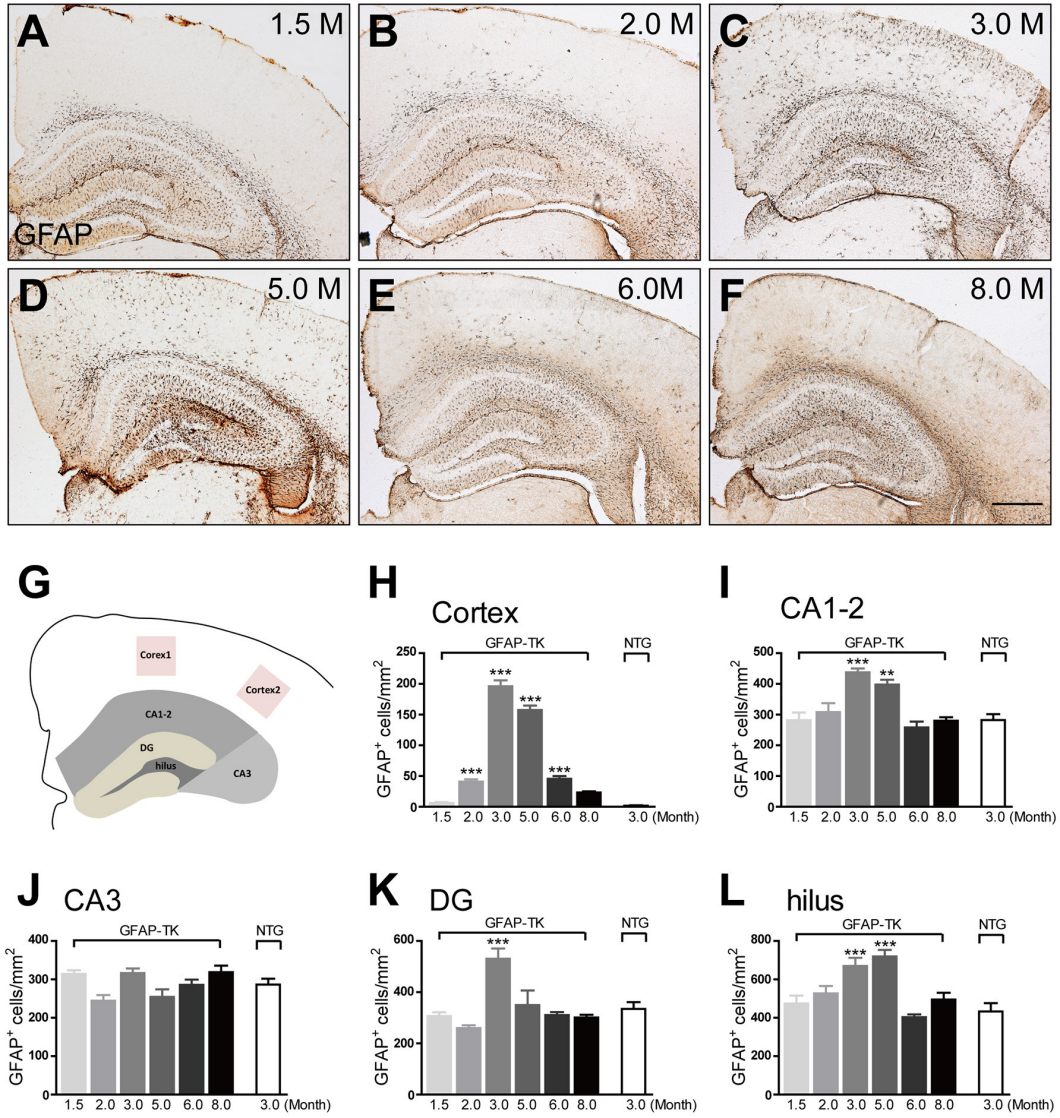
**The increased expression of GFAP in the cortex and hippocampus of 3-month-old TK-1 mice was found between 3 and 5 months old of age (A–F)** Representative photomicrographs of GFAP^+^ cells in the cortex and hippocampus of 1.5-, 2-, 3-, 5-, 6-, and 8-month-old TK-1 mice. Scale bar, 500 μm. **(G)** Schematic depicting regions of the cortex and hippocampus analyzed for this study. **(H)** Quantification of the number of GFAP^+^ cells in the cortex of 1.5-, 2-, 3-, 5-, 6-, and 8-month-old TK-1 mice (*n* = 3 mice per group, 3 brain slices per mouse). **(I–L)** Quantification of the number of GFAP^+^ cells in the hippocampus of 1.5-, 2-, 3-, 5-, 6-, and 8-month-old TK-1 mice (*n* = 3 mice per group, 3 brain slices for each mouse). **(I)**, CA1-2; **(J)**, CA3; **(K)**, DG; **(L)**, hilus. Data represent mean ± SEM,^*^P < 0.05, ^**^P < 0.01, ^***^P < 0.001 (one-way ANOVA with *post hoc* Turkey's multiple comparison test).

To determine whether astrocytes were affected in the brain of GFAP-TK mice, TK-1, and TK-2 mice at different ages were used for staining of markers for astrocytes. Our results showed that the number of GFAP- and vimentin-positive astrocytes was dramatically increased in the cortex and hippocampus of 3-month-old TK-1 mice, but not in the TK-2 mice (Figures 2A–C). Western blot analysis confirmed that the expression of GFAP and vimentin was greatly increased in the hippocampus of 3-month-old TK-1 mice compared with that of both age-matched controls and TK-2 mice (Figures 2D–F). Interestingly, the increased expression of GFAP in the cortex and hippocampus of TK-1 mice was only found between 3 and 5 months old of age (Figure 3).

### The expression of iba1 was not changed in TK-1 and TK-2 mice

**Figure 4:**
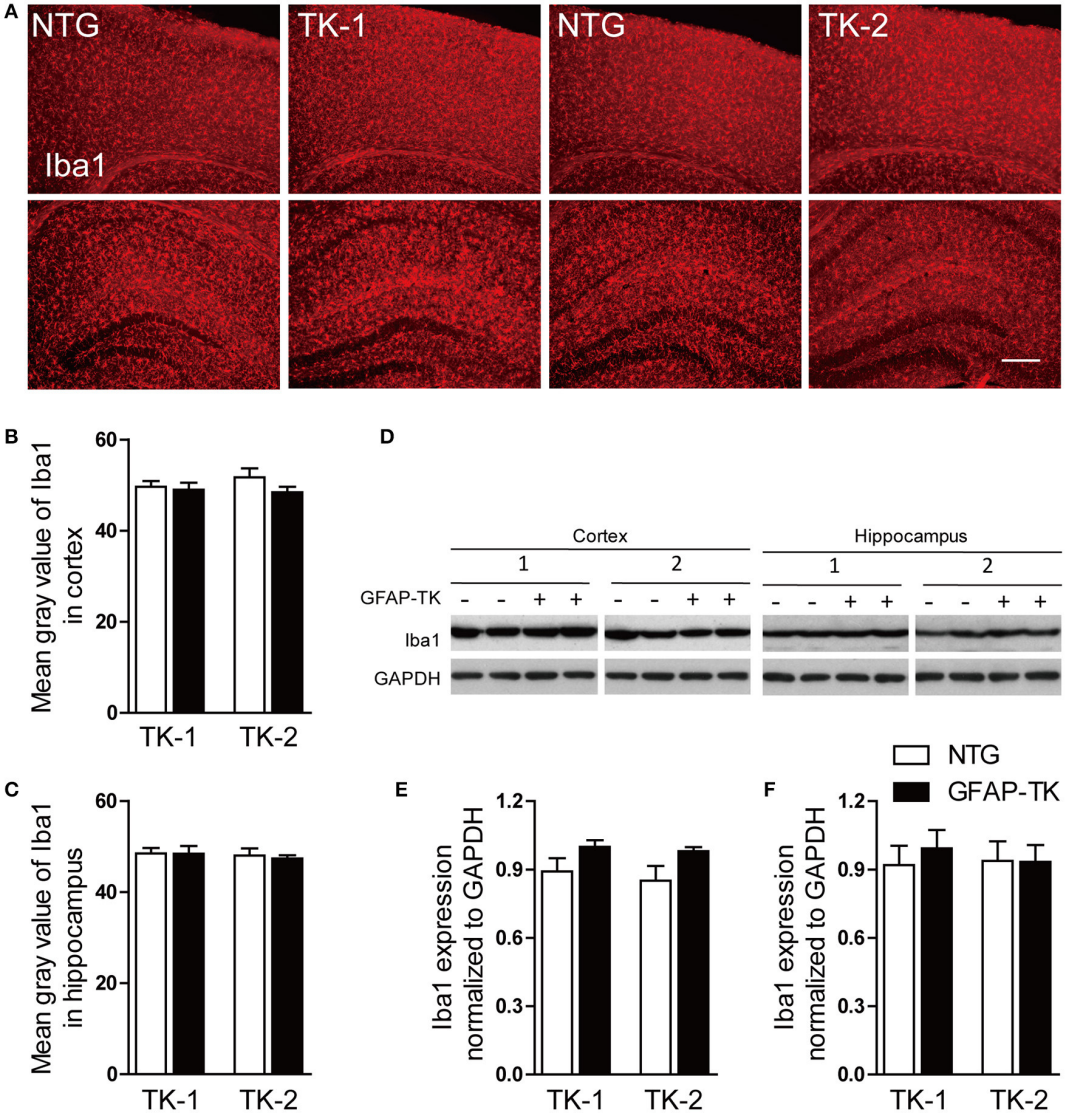
**The expression of Iba1 was not affected in the cortex and hippocampus of both TK-1 and TK-2 mice. (A)** Representative photomicrographs of Iba1^+^ cells in the cortex and hippocampus of 3-month-old TK-1 and TK-2 mice and their age-matched controls. Scale bar, 200 μm. **(B,C)** Fluorescence intensity was quantified by using image-analysis software in the cortex and hippocampus. (*n* = 3 mice per group, 3 brain slices for each mouse). **(D)** Protein bands of Iba1 in the cortex and hippocampus, GAPDH severed as the loading control. **(E)** Quantification of the level of Iba1 in the cortex of 3-month-old TK-1 (*n* = 4 mice) and TK-2 mice (*n* = 4 mice) and their age-matched controls (*n* = 4 mice per group). **(F)** Quantification of the level of Iba1 in the hippocampus of 3-month-old TK-1 (*n* = 4 mice) and TK-2 mice (*n* = 4 mice) and their age-matched controls (*n* = 4 mice per group). Data represent mean ± SEM.

To determine whether microglia was affected in GFAP-TK mice, we examined the expression of Iba1 by immunostaining and western blotting in the cortex and hippocampus of 3-month-old TK-1 and TK-2 mice and their age-matched controls. No difference of Iba1 expression was found in the hippocampus and cortex of TK-1 and TK-2 mice compared with their controls (Figure 4), suggesting that microglia were not affected in the GFAP-TK mice.

### The expression of NeuN and MAP2 was not affected in both TK-1 and TK-2 mice

**Figure 5:**
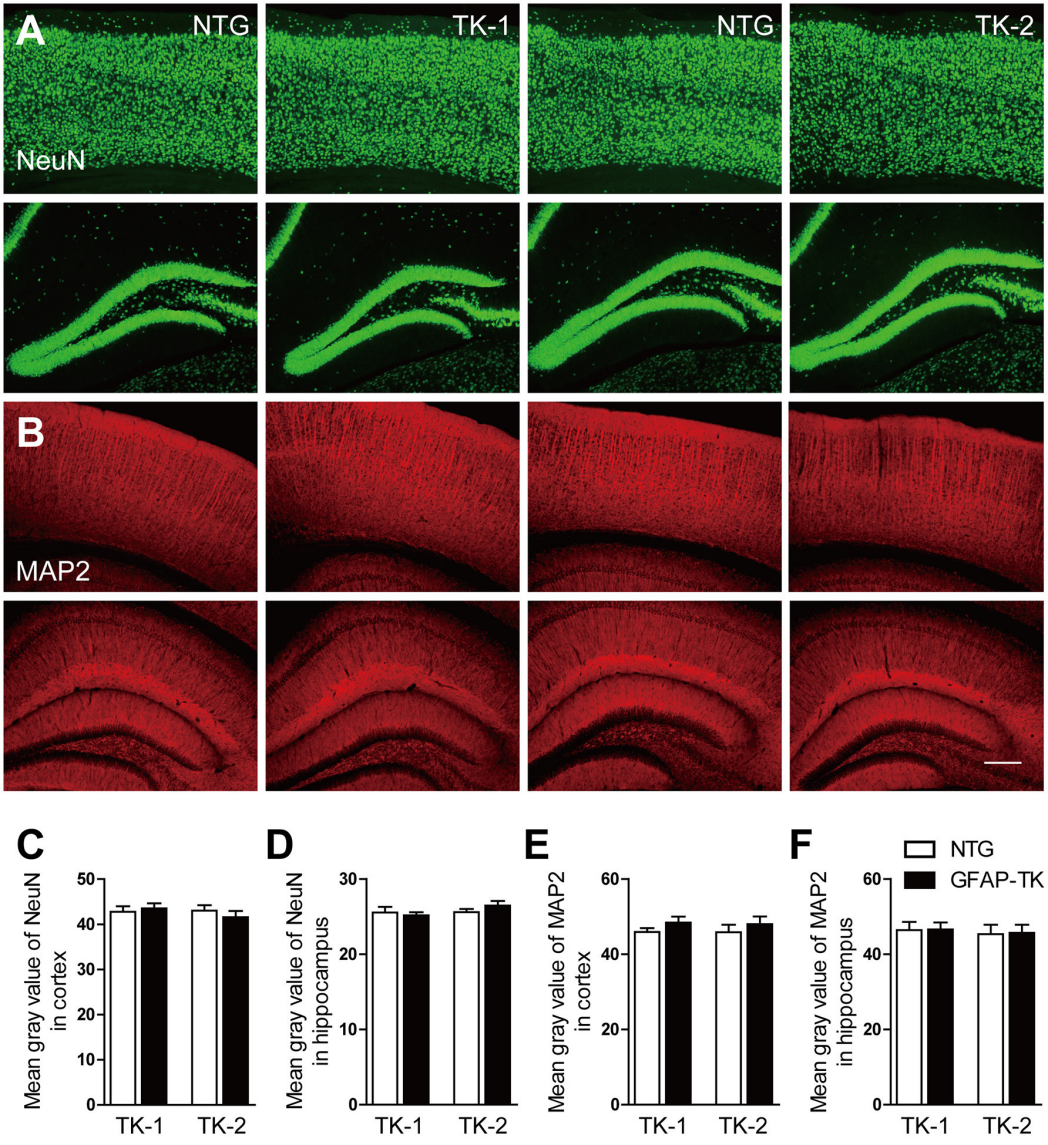
**The expression of NeuN and MAP2 was not affected in the cortex and hippocampus of both TK-1 and TK-2 mice (2). (A)** Representative photomicrographs of NeuN^+^ cells in the cortex and hippocampus of 3-month-old TK-1 and TK-2 mice and their age-matched controls. **(B)** Representative photomicrographs of MAP2^+^ cells in the cortex and hippocampus of 3-month-old TK-1 and TK-2 mice and their age-matched controls. Scale bar, 200 μm. **(C,D)** Quantification of the fluorescence intensity of NeuN positive signals in the cortex and hippocampus. (*n* = 3 mice per group, 3 brain slices for each mouse). **(E,F)** Quantification of the fluorescence intensity of MAP2 positive signals in the cortex and hippocampus. (*n* = 3 mice per group, 3 brain slices for each mouse). Data represent mean ± SEM.

To determine whether neurons were affected in GFAP-TK mice, we checked the expression of NeuN and MAP2 by immunofluorescent staining. No difference in NeuN and MAP2 expression was found in the hippocampus and cortex of both TK-1 and TK-2 mice (3-month-old) compared their age-matched controls (Figure 5), suggesting that neurons were not affected in the GFAP-TK mice.

### The expression of AQP4 was increased in TK-1 mice

**Figure 6:**
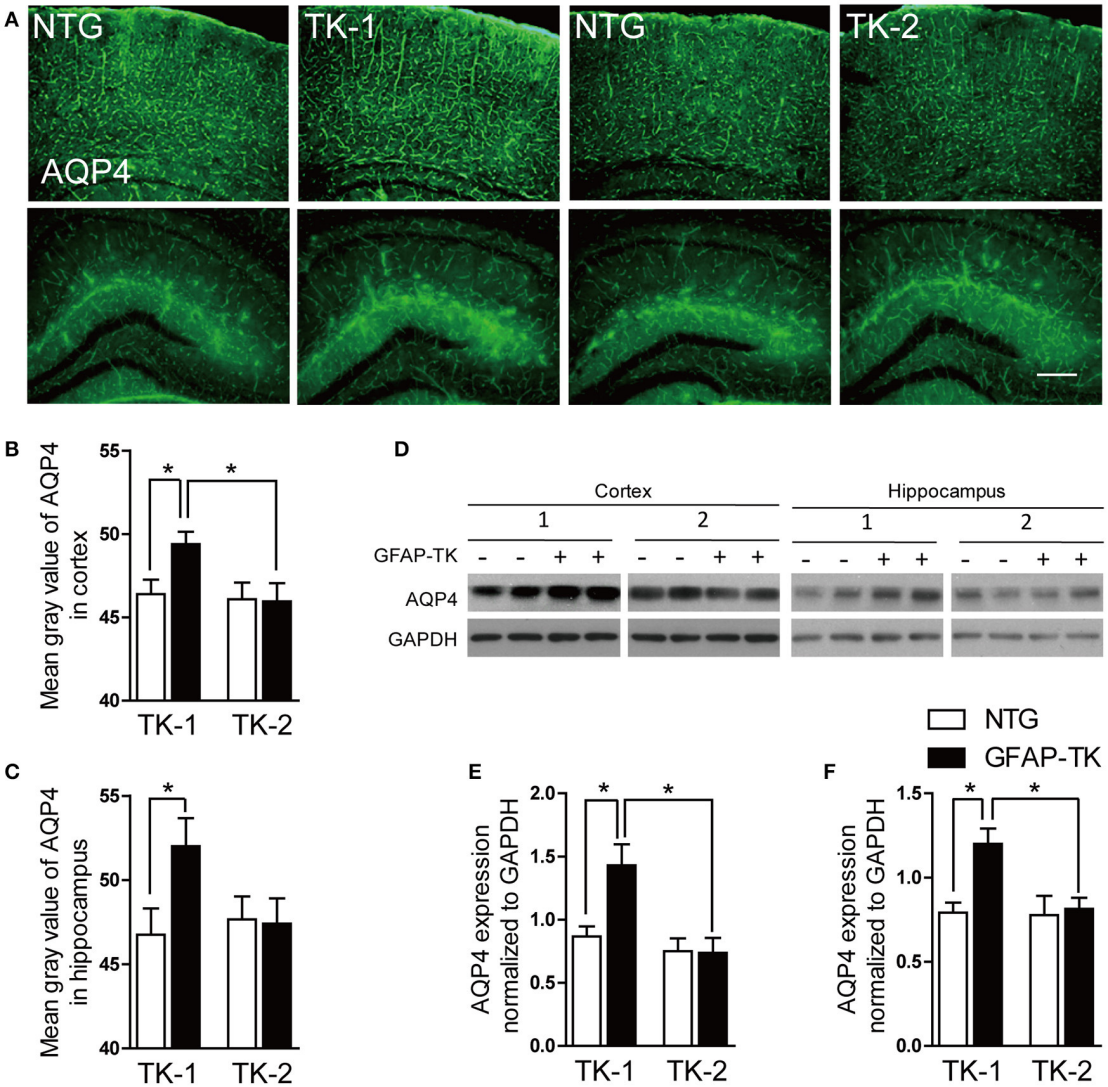
**The expression of AQP4 was increased in the cortex and hippocampus of 3-month-old TK-1 mice. (A)** Representative photomicrographs of AQP4 expression in the cortex and hippocampus of 3-month-old TK-1 and TK-2 mice and their age-matched controls. Scale bar, 200 μm. **(B,C)** Fluorescence intensity was quantified by using image-analysis software in the cortex and hippocampus. (*n* = 3 mice per group, 3 brain slices for each mouse). **(D)** Protein bands of AQP4 in the cortex and hippocampus, GAPDH severed as the loading control. **(E)** Quantification of the level of AQP4 in the cortex of 3-month-old TK-1 (*n* = 7 mice) and TK-2 mice (*n* = 4 mice) and their age-matched controls (*n* = 4 mice per group). **(F)** Quantification of the level of AQP4 in the hippocampus of 3-month-old TK-1 (*n* = 7 mice) and TK-2 mice (*n* = 4 mice) and their age-matched controls (*n* = 4 mice per group). Data represent mean ± SEM, ^*^P < 0.05 (unpaired *t*-test).

AQP4 is abundantly expressed in astrocytic vascular end-feet and plays important roles in regulating the physiological functions of astrocytes (Nedergaard, 2013). To determine whether AQP4 expresison was affected in the GFAP-TK mice, we performed immunofluorescent staining and western blot to examine the expression of AQP4. As a result, the expression of AQP4 was increased in the cortex and hippocampus of 3-month-old TK-1 mice compared with that of both age-matched controls and TK-2 mice (Figure 6).

## Discussion

Several lines of GFAP-TK mice have been developed and they were widely used in studies on neurogenesis and reactive astrocytes (Garcia et al., 2004; Snyder et al., 2005). Previous studies reported that GCV treatment in GFAP-TK mice resulted in reduced neurogenesis and deletion of proliferating GFAP-expressing astrocytes without affecting mature GFAP-expressing astrocytes (Garcia et al., 2004; Lepore et al., 2008). In the present study, we found that GCV treatment effectively inhibited the neurogenesis in the adult hippocampus of the GFAP-TK mice purchased from the Jackson Laboratory (Stock No. 005698). However, GFAP- and vimentin-expressing astrocytes were dramatically increased in the cortex and hippocampus of this line of GFAP-TK mice with or without GCV treatment, suggesting that both adult neural stem cells and mature astrocytes were affected. In a second line of GFAP-TK mice (MMRRC, Stock No. 037351-UNC) generated in Dr. Heather Cameron's laboratory in NIH (Snyder et al., 2011), GCV treatment induced dramatic depletion of DCX-positive cells in the dentate gyrus of adult GFAP-TK^+^ mice, suggesting effective inhibition of adult neurogenesis. However, no difference of GFAP and vimentin expression was observed in both hippocampus and cortex between GFAP-TK^+^ and GFAP-TK^−^ mice, regardless of GCV treatment or not, suggesting that mature astrocytes were not affected in this line of GFAP-TK mice. Although we did not have an explanation for the difference mentioned above, the different genetic background of the two lines of mice could be one of the reasons, and our data suggested that the GFAP-TK mice (MMRRC, Stock No. 037351-UNC) generated in Dr. Heather Cameron's laboratory were better than the GFAP-TK mice purchased from the Jackson Laboratory (Stock No. 005698) for exploring the roles of adult neurogenesis.

## Author contributions

XZ, DW, and HP performed all experiments; XZ, DW, and BS processed and analyzed all data; and XZ, and BS wrote the manuscript; BS supervised the study.

### Conflict of interest statement

The authors declare that the research was conducted in the absence of any commercial or financial relationships that could be construed as a potential conflict of interest.

## Abbreviations

GFAP: glial fibrillary acidic protein
AQP4: aquaporin 4
HSV-TK: herpes simplex virus thymidine kinase
GCV: ganciclovir
MAM: Methylazoxymethanol acetate
DCX: doublecortin
DG: dentate gyrus
SGZ: subgranular zone.
